# Urbanization and physician maldistribution: a longitudinal study in Japan

**DOI:** 10.1186/1472-6963-11-260

**Published:** 2011-10-08

**Authors:** Shinichi Tanihara, Yasuki Kobayashi, Hiroshi Une, Ichiro Kawachi

**Affiliations:** 1 Department of Preventive Medicine and Public Health, Faculty of Medicine, Fukuoka University, Fukuoka, Japan; 2 Department of Public Health, Graduate School of Medicine, The University of Tokyo, Tokyo, Japan; 3 General Medical Research Center, Faculty of Medicine, Fukuoka University, Fukuoka, Japan; 4 Department of Society, Human Development, and Health, Harvard School of Public Health, Boston, MA, USA

## Abstract

**Background:**

The relative shortage of physicians in Japan's rural areas is an important issue in health policy. In the 1970s, the Japanese government began a policy to increase the number of medical students and to achieve a better distribution of physicians. Beginning in 1985, however, admissions to medical school were reduced to prevent a future oversupply of physicians. In 2007, medical school entrants equaled just 92% of their 1982 peers. The urban annual population growth rate is positive and the rural is negative, a trend that may affect denominator populations and physician distribution.

**Methods:**

Our data cover six time points and span a decade: 1998, 2000, 2002, 2004, 2006, and 2008. The spatial units for analysis are the secondary tier of medical care (STM) as defined by the Medical Service Law and related legislation. We examined trends in the geographic disparities in population and physician distribution among 348 STMs in Japan. We compared populations and the number of physicians per 100,000 populations in each STM. To measure maldistribution quantitatively, we calculated Gini coefficients for physician distribution.

**Results:**

Between 1998 and 2008, the total population and the number of practicing physicians for every 100,000 people increased by 0.95% and 13.6%, respectively. However, the inequality of physician distribution remained constant, although small and mostly rural areas experienced an increase in physician to population ratios. In contrast, as the maldistribution of population escalated during the same period, the Gini coefficient of population rose. Although the absolute number of practicing physicians in small STMs decreased, the fall in the denominator population of the STMs resulted in an increase in the number of practicing physicians per population in those located in rural areas.

**Conclusions:**

A policy that increased the number of physicians and the physician to population ratios between 1998 and 2008 in all geographic areas of Japan, irrespective of size, did not lead to a more equal geographical distribution of physicians. The ratios of physicians to population in small rural STMs increased because of concurrent trends in urbanization and not because of a rise in the number of practicing physicians.

## Background

A relative shortage of physicians in rural areas has been reported over the last few decades in Japan [1-5]. In the 1970s, the Japanese government introduced the policy of establishing at least one medical school per prefecture and increasing the total number of medical students. By the mid-1980s, the number of medical graduates, or newly certified physicians, per year had doubled from about 4000 to 8000. In 1984, the Ministry of Health and Welfare (now the Ministry of Health, Labour and Welfare and hereafter MHLW) announced that the targeted ratio of physicians per population had been achieved. As a result, beginning in 1985, medical school admissions were reduced to prevent a future oversupply of physicians. In 1993, the numbers of those entering medical schools fell to 7725, or 7.7% fewer than in 1984. Since the target reduction was 10% from the number admitted in 1984, the cutback in entering medical students continued. In 1997, a cabinet meeting approved this further reduction. In 2007, 7625 students were accepted for medical training, or 92% of the 1982 entering class.

However, by 2007, a relative shortage of physicians in Japan became evident. In that year, the number of physicians per 1,000 population was 2.1, while the number of medical graduates per population of 100,000 was 6.0, ratios that placed the nation in the 26^th ^and 28^th ^positions respectively among the 30 Organisation for Economic Co-operation and Development (OECD) countries [6]. On May 31, 2007, the Japanese government announced the "Active Plan for urgent supply of physician." From 2007 to 2008, the Ministry of Education, Culture, Sports, Science and Technology, increased medical school admissions for the first time in twenty-eight years, permitting 7793 students to begin their training. The increase in new medical students continued, and by 2009, their numbers reached 8,486, exceeding the peak of the early 1980's. In 2010, 8,846 were admitted, a 16% increase from the low point at the beginning of the century.

Previous studies of this increase in medical students on the distribution of physicians in Japan reveal that while the absolute shortage of physicians lessened, their geographical maldistribution persisted [1,4,5]. Furthermore, since 2004, MHLW has implemented mandatory two-year post-graduate training in designated clinical training hospitals for all newly certified physicians. Inequalities in physician distribution may have widened after the implementation of this mandatory system, since most clinical training facilities are either university hospitals or those certified for training by the MHLW, and both types are typically located in urban areas. However, few studies exist on the impact of the new post-graduate training system on physician distribution in Japan [4].

To analyze disparities in the allocation of physicians, the number of physicians per population at the municipality or county level is commonly used [1,3-5,7,8]. However, a number of published analyses suggest that this indicator is not necessarily useful or revealing at the municipality level in Japan, for while the populations of municipalities vary by an order of magnitude of 5000 in Japan [9], it assumes that residents only seek care in their own county and ignore patient flows into adjacent areas [8,10,11]. The proportion of municipalities with small populations is high in Japan. In 1999, 1531 (45.5%) of 3368 municipalities in the nation contained fewer than 10,000 people. After a massive merger of municipalities undertaken between 1998 and 2006, the total number of municipalities decreased, as did the proportion of those with small populations. By 2009, Japan had 1926 municipalities of which 489 (25.4%) had populations smaller than 10,000. Because of the high proportion of small municipalities, the percentage of these with small numbers of practicing physicians is high. Surveys of physicians, dentists, and pharmacists conducted by the MHLW in 1998 indicated that 1711 of 3371 municipalities (50.8%) had nine or fewer practicing physicians. After the period of municipal mergers between 1998 and 2006, 557 of 1951 municipalities (29.6%) had nine or fewer practicing physicians in 2008.

This finding indicates that many municipalities might be too small to maintain medical facilities employing multiple physicians. In other words, examining medical resource allocation at the municipal level does not necessarily yield insightful information. Consequently, in analyzing physician allocation disparities, it is best to define a region as an entity whose population is larger than those of counties and municipalities and smaller than those of states, provinces, and prefectures. In dealing with large states, this approach assumes that the physician per population ratio is uniform throughout the state, even if persons living in areas adjacent to metropolitan counties are more likely to seek care in the latter [8,12-17].

The 1985 revisions to the Medical Service Law (here after the Medical Service Law) directed each prefecture to establish a system of regional medical services, in order to provide efficient and appropriate medical care with finite resources and to improve collaboration between medical, community health, and social welfare service providers. The primary tier of medical care is mainly concerned with primary care and ordinary outpatient care. The Medical Service Law contains no specific regulations for the spatial unit of this primary tier. However, in most prefectures, the municipality is its spatial unit. The secondary tier of medical care (STM) is mainly concerned with most admissions and surgical, emergency, and ambulatory services. Usually, a prefecture is divided into five to ten STMs on the basis of its medical resources, transportation, and geographical situation. Most of the STMs are based on a complex of adjacent municipalities. Therefore, the STM is concerned with primary care, ordinary and specific outpatient care, and usual inpatient care. "Initial, secondary and tertiary emergency medical services" are all contained within a STM. Inflows and outflows of patients are very limited in a STM, except for specific and advanced treatment of certain special conditions. According to the Patient Survey [18], 76% of the hospitalized patients lived in the same STM of the hospital in 2008. The tertiary tier of medical care provides medical services for an area that cannot readily be met by the primary and secondary tiers, such as the treatments performed at a university hospital.

After 1950, although annual population growth rate was positive in the urban areas of developed countries, including Japan, it was negative in rural ones [19]. Local population decline and the inflow of people to urban areas continue. In considering the number of the physicians per population, we must examine the change of both population and the number of the physicians at the same time because urbanization widens the disparity between the rural area and urban areas. Particularly in areas with a small number of inhabitants, the number of the physicians per population increases when the percentage of population decline exceeds that of physician decrease. In this case, it may be difficult to maintain the function of medical facilities, such as hospitals, because the number of the physicians actually falls.

As mentioned above, the STM is an area established by the Medical Service Law and related legislation. However, few inquiries into the allocation of medical resources at the STM level have been carried out [20,21]. In the study presented here, we examine three hypotheses. First, the increase in the number of physicians between 1998 and 2008 did not lead to a more equal distribution of physicians. Second, during the same period, the maldistribution of population escalated. Third, despite the decrease in the number of practicing physicians in STMs with smaller numbers of inhabitants, the fall in the denominator populations caused an increase in the former per population.

## Methods

Our data cover six time points and span a decade: 1998, 2000, 2002, 2004, 2006, and 2008. Data on the number of physicians were obtained from surveys conducted by the MHLW. By law, physicians in Japan must report their places of work, specialties, and facilities every two years, and since 1977, the MHLW has been publishing data on the number of physicians practicing in each municipality. Physicians who reported that their principal activity was non-clinical (medical research, education, government administration, and so on) were excluded from the analysis. Data on the number of physicians were obtained from Survey of Physicians, Dentists, and Pharmacologists [22]. Data on municipal populations were obtained from the Basic Resident Registers collected and compiled by the Ministry of Internal Affairs and Communications in March of each year [9].

Between 1998 and 2009, a massive merger of municipalities occurred. The total number of municipalities decreased from 3,232 to 1,804 between 1998 and 2009. The total number of STMs did not change significantly, but their borders were frequently redrawn. To examine the trend in the geographic distribution of physicians using the Gini coefficient, the number and boundaries of geographic units must be fixed. Therefore, we converted the boundaries of STMs in 1998, 2000, 2002, 2004 and 2006 into the boundaries of 2008. Then, we adapted the municipality data from the first five years into that of the sixth.

We compared populations and the number of physicians per 100,000 population in each STM. To measure maldistribution quantitatively, we used the maximum/minimum ratios, the 90 percentile/10 percentile ratios, the top quartile/third quartile ratios, and the Gini coefficient. The Gini coefficient has been most commonly used to measure inequality in the distribution of incomes. It is derived from the Lorenz curve, which is based on a curve fitted to percentile shares of income and population. Several research groups have applied this method to study physician distribution [1,4,5]. To draw the Lorenz curve, we plotted all the points representing the percentile shares of practicing physicians and population. Finally, the Gini coefficients for physician distribution in 1998, 2000, 2002, 2004, 2006, and 2008 were calculated. Next, we examined the change in the absolute number of practicing physicians and the number of practicing physicians per populations of 100,000 between 1998 and 2008 among STMs on the basis of 1998 populations. These statistical analyses were done with Stata software, version 10.1 (StataCorp LP, College Station, TX, USA).

## Results

Table 1 shows the trend in the number of practicing physicians per 100,000 people in Japan from 1998 to 2008. This figure increased from 236,933 in 1998 to 271,897 in 2008, or 14.8%. The total population increased 0.95% and the number of practicing physicians per 100,000 increased 13.6%.

**Table 1:** Trend in the number of practicing physicians per populations of 100,000 in Japan

Year	1998	2000	2002	2004	2006	2008
Total population (million)	126.0	126.3	126.7	126.9	127.1	127.1
The number of practicing physicians	236,933	243,201	249,574	256,668	263,540	271,897
Practicing physicians per 100,000 population	188.1	192.6	197.0	202.3	207.4	214.0

Table 2 indicates the trend in the distribution of populations in the STMs from 1998 to 2008. The maximum population of the STMs was about 2,500,000 in these years. Overall, there was a bipolar trend in populations of STMs: more heavily populated ones slightly increased their populations from 1998 to 2008; however, those with relatively smaller populations experienced the opposite tendency. All of the indexes reveal that the inequality of populations among STMs escalated in these years.

**Table 2:** Trend in the distribution of population in the secondary tier of medical care in Japan

Year	1998	2000	2002	2004	2006	2008
Max	2,472,294	2,474,579	2,490,172	2,497,208	2,510,459	2,525,153
90 Percentile (P90)	830,718	834,303	838,740	838,811	835,040	837,077
Top Quartile (Q1)	463,264	465,518	465,045	466,522	466,373	464,016
Median	225,062	224,677	223,460	222,321	220,902	218,623
Third quartile (Q3)	108,224	106,822	105,431	103,719	101,833	99,238
10 Percentile (P10)	71,421	70,143	68,819	67,296	65,678	64,051
Min	25,898	25,228	24,807	24,106	23,347	22,466
Max/Min	95.5	98.1	100.4	103.6	107.5	112.4
P90/P10	11.6	11.9	12.2	12.5	12.7	13.1
Q1/Q3	4.28	4.36	4.41	4.50	4.58	4.68
Gini coefficient	0.497	0.499	0.503	0.506	0.510	0.515
95% confidence interval	0.467-0.527	0.469-0.529	0.472-0.533	0.476-0.536	0.480-0.540	0.485-0.545

Table 3 shows the change of the populations of STMs between 1998 and 2008, according to population size in the baseline year. Of 348 STMs, those with populations between 100,000 and 200,000 were most numerous, accounting for approximately a quarter the total. The second largest group, those with populations of less than 100,000, accounted for approximately a quarter of all STMs.

**Table 3:** Change in the populations of the secondary tier of medical care between 1998 and 2008 relative to population size of 1998

Population size in 1998	Change of the population between 1998 and 2008		
	
	Decreased	Increased	Total
< 100,000	75 (96%)	3 (4%)	78 (100%)
100,000-199,999	71 (90%)	8 (10%)	79 (100%)
200,000-299,999	43 (78%)	12 (22%)	55 (100%)
300,000-399,999	23 (68%)	11 (32%)	34 (100%)
400,000-499,999	12 (46%)	14 (54%)	26 (100%)
500,000-999,999	12 (23%)	40 (77%)	52 (100%)
1,000,000≤	3 (12%)	21 (88%)	24 (100%)
Total	239 (69%)	109 (31%)	348 (100%)

The smaller the population of the STMs, the higher is the percentage with demographic decline. Among 78 STMs with fewer than 100,000 people, 75 (96%) lost population. Among the 24 STMs with populations of more than one million, only 3 (12%) STMs experienced such a decrease. The tendency for STMs to lose inhabitants according to population size is statistically significant (p < 0.001). Thus, a continuous migration from STMs with small populations to those with large ones took place.

Table 4 shows the trends in the distribution of practicing physician per populations of 100,000 by STMs. The maximums were between 1,100 and 1,200 with no definite trend throughout the study period, while the minimum showed a constant increase. As a result, the ratio of maximum to minimum decreased. Because the lower limit of the top quartile and that of the third quartile increased at almost the same pace over the study period, the ratios of top quartile/third quartile through 1998 to 2008 were between 1.47 and 1.51 respectively, without any specific direction. The Gini coefficient changed little, staying between 0.21 and 0.22 for the years studied.

**Table 4:** Trend in the number of practicing physicians per populations of 100,000 in the secondary tier of medical care in Japan

Year	1998	2000	2002	2004	2006	2008
Max	1190.0	1191.1	1136.1	1164.3	1145.4	1177.7
90 Percentile (P90)	249.2	255.6	259.4	265.8	271.0	278.7
Top Quartile (Q1)	186.3	192.0	197.6	201.0	203.3	206.4
Median	150.1	154.7	157.4	162.1	162.1	165.2
Third quartile (Q3)	123.3	130.4	133.2	134.8	138.7	139.4
10 Percentile (P10)	105.7	108.9	114.9	114.2	114.7	116.9
Min	55.8	57.0	59.9	64.0	71.7	71.7
Max/Min	21.3	20.9	19.0	18.2	16.0	16.4
Q1/Q3	1.51	1.47	1.48	1.49	1.47	1.48
P90/P10	2.36	2.35	2.26	2.33	2.36	2.38
Gini coefficient	0.224	0.219	0.215	0.213	0.211	0.214
95% confidence interval	0.185-0.263	0.180-0.258	0.178-0.251	0.175-0.252	0.173-0.249	0.175-0.253

Table 5 displays the change in the absolute number of practicing physicians and the number of practicing physicians per populations of 100,000 between 1998 and 2008 among STMs according to population size in 1998. Among the 348 STMs, the absolute number of practicing physicians decreased in 97 (28%) and remained unchanged in 5 (1%). Of these, 55 (54%) increased the number of practicing physicians per populations of 100,000. Thus, as the populations of STMS decreased so did the number of physicians serving in them. Of 348 STMs, 50 (15%) witnessed a fall in the number of practicing physicians per populations of 100,000. The tendency for this decline to occur according to the size of populations was significantly high (p < 0.001). All the STMs with 1,000,000 or more people gained more practicing physicians, both absolutely and in terms of each 100,000 inhabitants.

**Table 5:** Change in the absolute number of practicing physicians and the number of practicing physicians per populations between 1998 and 2008 relative to population size of 1998

The change for the absolute number of practicing physicians (A) and the number of practicing physicians per populations (B)					
**Population size in 1998**	**Both decreased or unchanged**	**(A) decreased or unchanged and (B) increased**	**(A) increased and (B) decreased or unchanged**	**Both increased**	**Total**

< 100,000	28 (36%)	24 (31%)	0 (0%)	26 (33%)	78 (100%)
100,000-199,999	9 (11%)	23 (29%)	1 (1%)	46 (58%)	79 (100%)
200,000-299,999	8 (15%)	7 (13%)	0 (0%)	40 (73%)	55 (100%)
300,000-399,999	1 (3%)	1 (3%)	0 (0%)	32 (94%)	34 (100%)
400,000-499,999	1 (4%)	0 (0%)	0 (0%)	25 (96%)	26 (100%)
500,000-999,999	0 (0%)	0 (0%)	2 (4%)	50 (96%)	52 (100%)
1,000,000≤	0 (0%)	0 (0%)	0 (0%)	24 (100%)	24 (100%)
Total	47 (14%)	55 (16%)	3 (1%)	243 (70%)	348 (100%)

## Discussion

In this study, we analyze the trends in the geographic disparities of population and physician distribution among STMs. We arrive at three major findings. First, the inequality in physician distribution has remained stable over the last decade. Second, the maldistribution of STM populations escalated from 1998 through 2008. Third, despite the decrease in the number of practicing physicians, the fall in the denominator populations caused an increase in the number of practicing physicians per populations in STMs with a small number of inhabitants (less than 200,000).

This study shows that the trends in geographic disparities in the allocation of physicians among STMs did not change during the last decade in Japan. Although the ratio of physicians per populations of 100,000 increased throughout the country, the inequality in physician distribution did not improve [1,5]. The maldistribution of physicians working in hospitals accelerated after the implementation of mandatory post-graduate training for all newly certified physicians [4]. However, the 1985 revisions to the Medical Service Law directed each prefecture to establish a system of regional medical services including the number of hospital beds in each STM. In other words, it is difficult for the municipalities with small populations to maintain medical facilities employing multiple physicians, and it is impossible to evaluate the allocation of medical care resources if the municipality is the spatial unit of analysis.

The maldistribution of people among STMs escalated from 1998 through 2008. Most of the STMs with large populations are located in the urban area, which grew in this period. Since 1950, while the urban areas of developed countries have had a positive annual growth rate, their rural areas have had a negative one [19]. Regional disparities of medical resource allocation has been reported many times [1,3-5,7,8,11-17]; however, the change in the socio-economic factors of spatial units has not been intensively examined before.

Because of decreasing rural populations, about one in five STMs with fewer than 200,000 people saw a relative increase in the numbers of practicing physicians, in spite of the absolute fall in the number of the latter. Even if the number of the physicians per population remains unchanged, their presence is actually limited in spatial units with small populations. It is desirable that every spatial unit has a minimum population scale for maintaining medical resources. Earlier studies that accept the municipality as the spatial unit [1,3-5] ignore the more than 5,000 time difference in scale between the smallest and largest. In this study, the difference of population among STMs is only about 100 times, thus, this earlier shortcoming is greatly mitigated. In areas where it is difficult to set a minimum population scale, such as a remote island, the evaluation of the allocation of medical resources is a vexing problem.

This study is unique because it focuses on the physician distribution among STMs, as defined by the 1985 revisions to the Medical Service Law. Previous studies of trends in geographic disparities in the allocation of physicians report that the gap between urban and rural areas has widened, despite an increase in the total number of physicians [1,4,5,7,8,13,23-25]. These studies measure the number of physicians per population by county [7,13,23] or municipality [1,4,5]. Physician to patient ratios by county are not necessarily revealing measures of access to physician services, since they unrealistically assume that residents only seek care in their own county; in fact patients in small counties may travel to see a physician in adjacent areas [8,12]. This approach also posits that in large, heavily-populated areas, the physician per population ratios are equal over the entire area, even though people living in areas adjacent to metropolitan counties are more likely to seek care in the latter [8,11,13]. Estimating the caseloads of physicians [8,17,24] and of travel time or distance to the nearest physician [17,26,27], whether those that offer primary [17] or specialist care [17,27] and to medical facilities [16,27,28] is important in defining the levels of equity and efficiency in a healthcare system.

Examining medical resources allocation at the municipal level is not necessarily helpful because of the high proportion of small municipalities. In 2009, 1926 municipalities existed, of which 489 (25.4%) had populations of 10,000 or fewer inhabitants. The surveys of physicians, dentists, and pharmacists conducted by the MHLW in 2008, reveal 1951 municipalities of which 557 (29.6%) had nine or fewer practicing physicians. Therefore, many municipalities might have been too small to maintain medical facilities employing multiple physicians. In Japan, the population of large cities were more than 5,000 times larger than small villages in 2009 (range 157-831,224) [9]. The population differences of the spatial units in Primary Care Service Areas in the United States [15] and Switzerland [17] are of the order of 1000 and that of the hospital service areas in Switzerland [16] of an order of 200.

This study has several limitations. The first is that socioeconomic factors, except the population scale, are not examined, although the populations of STMs vary by a magnitude of about 100. Earlier studies report that socioeconomic factors affect physician allocations [5,16,17,23,29]. The proportion of the population residing in urban agglomerations will persist into the future, and the disparity of socioeconomic factors will widen. A further examination of the definition of a region used in the analysis of disparities in physician allocation is necessary. Furthermore, we do not consider the experience or specialty of physicians. Our study includes data that aggregate all practicing physicians. The distinction between generalists and specialists remains obscure in Japan. Because free access is guaranteed in Japanese health insurance system, and patients are not required to see primary care physicians before consulting specialists, they can directly consult the latter. Future studies of physician specialties [3,17,27,29], the factors related to physician caseloads [8,17,24], and the relation between the increase of medical students and the change of physician allocations [1,4,5] would be helpful in identifying relatively underserved areas.

## Conclusions

There has been a substantive increase in the number of physicians at the national level in Japan. However, the 15% increase in the number of physicians between 1998 and 2008 did not lead to an alteration in physician distribution. The greater number of these medical providers was equally distributed across geographic areas. During the same period, however, the maldistribution of population escalated. The contraction of rural populations has led to an increase in the number of practicing physicians per population, despite the decrease of the latter in STMs with a small number of inhabitants (less than 200,000). Because of urbanization and population shifts away from those communities, the remaining residents benefited from increases in physician to population ratios, although they had no more doctors. Inequalities in health care resources must continue to be monitored.

## Abbreviations

STM: secondary tier of medical care; MHLW: Ministry of Health, Labour and Welfare

## Competing interests

The authors declare that they have no competing interests.

## Authors' contributions

ST conceived and coordinated the study, participated in its design, contributed to data analysis and drafted the manuscript; YK participated in study design, developed the statistical analysis model, and helped draft the manuscript; HU participated in study design and data collection; IK participated in study design and helped draft the manuscript; All authors approved the final version of the paper.

## Pre-publication history

The pre-publication history for this paper can be accessed here:

http://www.biomedcentral.com/1472-6963/11/260/prepub
